# Formation and Characterization of Self-Assembled Rice Protein Hydrolysate Nanoparticles as Soy Isoflavone Delivery Systems

**DOI:** 10.3390/foods12071523

**Published:** 2023-04-04

**Authors:** Haoran Mo, Xiuwen Chen, Bo Cui, Yangling Chen, Maolong Chen, Zhou Xu, Li Wen, Yunhui Cheng, Ye Jiao

**Affiliations:** 1 School of Food Science and Bioengineering, Changsha University of Science & Technology, Changsha 410114, China; 2 School of Food Science and Engineering, Qilu University of Technology, Shandong Academy of Sciences, Jinan 250353, China

**Keywords:** soy isoflavone, rice protein, nanoparticles transport systems

## Abstract

In this study, soy isoflavones-loaded nanoparticles were prepared using rice proteins (RPs) hydrolyzed by four types of enzyme (alcalase, neutrase, trypsin, and flavorzyme). After optimizing the preparation conditions, the encapsulation efficiency (EE) of the nanoparticles ranged from 61.16% ± 0.92% to 90.65% ± 0.19%. The RPs that were hydrolyzed by flavorzyme with a molecular weight of <5 KDa showed better characters on the formation of nanoparticles, and the formed nanoparticles had the highest EE and loading capacity (9.06%), the smallest particle size (64.77 nm), the lowest polymer dispersity index (0.19), and the lowest zeta potential (−25.64 mV).The results of Fourier transform ion cyclotron resonance, X-ray diffraction, and fluorescence spectroscopy showed that the nanoparticles were successfully encapsulated. The study of interaction showed that the formation of nanoparticles may depend mainly on hydrogen bonds, but other interactions, such as hydrophobic interactions and electrostatic interactions, cannot be ignored. After encapsulation, the pH stability, temperature stability, ionic stability, and oxidation resistance of the nanoparticles were enhanced. Moreover, the in vitro release experiment showed that the encapsulated nanoparticles had a certain protective effect on soybean isoflavones. In summary, rice protein hydrolysates are promising carriers for soybean isoflavones.

## 1. Introduction

Soy isoflavones (SIF) are the main secondary metabolites extracted from soybean and their derivative products [1]. SIF have been proven to have antioxidant activities due to their inherent phenolic hydroxyl, which can inhibit the free radicals produced in the human body [2]. Other functionalities, such as improvements in bone mineral density [3], remission of menopausal symptoms [4], and anticancer effects [5], have also been reported in the literature. However, SIF have low solubility and are sensitive to oxygen and light, which seriously affect their bioavailability [6,7]. SIF contents are affected by storage and processing conditions, and SIF contents and the respective compound pattern are subject to change upon thermal exposure [8]. All of those propose challenges for developing functional foods to improve human health.

Nanoencapsulation is a strategy in recent years for the protection of food ingredients and nutraceuticals and improve their stability, bioavailability and bioactivity [9]. In addition, nano-delivery systems can achieve the targeted delivery of bioactive molecules [10]. As a commonly used carrier material in natural polymer materials, protein has irreplaceable advantages in self-assembly capability and biocompatibility. Rice protein (RP) is a valuable plant protein due to its rich essential amino acids, proper amino acid ratio, and hypoallergenic properties compared with other cereals and legume proteins [11]. However, the poor solubility of RP at neutral pH limits its application [12]. Enzymatic hydrolysis is an effective means of improving the properties of RP [13], and rice protein hydrolysate (RPH) has great potential in the application of nanocarrier systems [14]. Enzymolysis can improve the functional properties and interfacial activity of the protein, while the peptides produced in the hydrolysis process often have many physiological activities with certain positive effects, such as anti-oxidation, anti-inflammatory, and anti-tumor activities. From the perspective of nutrition, protease hydrolysates can bring health effects without affecting their use as transport carriers [15]. Chen et al. [16] found that the rice protein based nanoemulsions prepared by RPH were shown a small droplet size (219.7 ± 2.1 nm) and good stability, and confirmed an effective carrier to deliver biologically active molecules. Besides, Ma et al. [17] reported that the solubility of lutein was improved by the construction of nanoparticles using the RPH (RP hydrolyzed by trypsin). However, the comprehensive evaluation of the properties of the nanoparticles formed by RPHs obtained by the hydrolysis of different proteases is limited.

This study systematically investigated the preparation, characterization, and functional evaluation of RPH-SIF nanoparticles. Four commercially available proteases were selected in this study: alcalase, neutrase, trypsin, and flavorzyme. The effects of the four enzymes on the structural and functional properties of RPH were evaluated by the changes in surface hydrophobicity, molecular weight distribution, and amino acid composition. Nanoparticles of soy isoflavone encapsulated by RPH were fabricated by the anti-solvent precipitation method. The encapsulation efficiency (EE) and loading capacity (LC) were used as indicators to evaluate the encapsulation properties. The RPH-SIF interactions were studied by fluorescence spectroscopy, X-ray diffraction (XRD), and infrared spectroscopy. The stability, antioxidant capacities and in vitro simulated digestion of the nanoparticles were investigated to highlight their potential applications as functional food or nutritional ingredients.

## 2. Materials and Methods

### 2.1. Materials

Rice was provided by Jinjian Cereals Industry Co., Ltd. (Changde, China). The RP was obtained in the laboratory using alkaline extraction and acid precipitation (protein content: 85% ± 0.25%). Soy isoflavone was purchased from Nanjing Spring and Autumn Biological Engineering Co., Ltd. (Nanjing, China). Alcalase, neutrase, flavorzyme, and trypsin were purchased from Beijing Solarbio Science and Technology Co., Ltd. (Beijing, China). All other reagents were of analytical grade and were purchased from Sinopharm Chemical Reagent Co., Ltd. (Shanghai, China). 

### 2.2. Preparation of Rice Protein Hydrolysates (RPHs)

The RP was hydrolyzed with alcalase, neutrase, flavorzyme, and trypsin, separately, at their optimum hydrolysis conditions (shown in Table S1) following the method reported by Xu et al. [14]. The RPHs prepared by hydrolysis with alcalase, neutrase, trypsin, and flavorzyme were named RPH(A), RPH(N), RPH(T), and RPH(F), respectively. The degrees of hydrolysis (DH, %) of RPH(A), RPH(N) and RPH(T) were determined using the pH-stat method [18], and the DH of RPH(F) was determined using the method reported by Xu et al. [14]. RPHs with different molecular weights (<3 KDa, <5 KDa, >5 KDa, Figure S1b) were separated using an MSC300 ultrafiltration cup (Sartorius Ltd., Ataturk, Germany), and then freeze-dried for preservation.

### 2.3. Characterization of RPHs

#### 2.3.1. Determination of the Molecular Weight Distribution

The molecular weight distributions of the RPHs were determined by high-performance liquid chromatography (HPLC, TSK gel 2000 SWXL, 300 mm × 7.8 mm). The mobile phase was water/acetonitrile/trifluoroacetic acid (90/10/0.1, *v/v/v*) and used isocratic washing. The sample was prepared with the above mobile phase to prepare a 1 mg/mL sample solution, which was passed through a 0.22 μm membrane for later use. The flow rate was 1 mL/min, and the column temperature was 30 °C. The detection wavelength was 220 nm.

#### 2.3.2. Measurement of the Surface Hydrophobicity (H_0_)

To determine the hydrophobicity (H_0_), 1-anilino-8-naphthalene sulphonate (ANS) were used to probe the hydrophobic fluorescence [19]. The RPH was dissolved in phosphate buffer (10 mM, pH 7.2), then the samples were diluted to 0.2, 0.4, 0.6, 0.8, and 1 mg/mL, respectively. An 8 mM solution of ANS in 10 mM phosphate buffer at pH 7.0 was prepared before the measurements. Protein solutions (4 mL) with various concentrations were thoroughly mixed with 20 μL of freshly prepared ANS. The mixtures were shaken vigorously and stored in the dark for 10 min. H_0_ was measured using a F-7100 fluorescence spectrophotometer (Hitachi Ltd., Tokyo, Japan). The instrument parameters were set as follows: excitation wavelength, 390 nm; emission wavelength, 470 nm; slit width, 5.0 nm. The H_0_ of the solution was calculated according to the fluorescence intensity of the series-diluted samples.

#### 2.3.3. Amino Acid Composition Analysis

The amino acid compositions of the RPHs were analyzed by HPLC (Venusil AA, Agela Ltd., Shanghai, China) using the method reported by Kuang et al. [20].

### 2.4. Preparation of RPH-SIF Nanoparticles

RPH-SIF nanoparticles were prepared by anti-solvent method [21]. SIF was dissolved in anhydrous ethanol at a concentration of 1 mg/mL, while RPH was dissolved in deionized water at the same concentration. 2 mL of SIF-ethanol was injected into 20 mL of RPH solution. The pH and temperature of the mixture were controlled at certain values (Table 1). Then, the mixture was stirred for 1 h and steamed using a rotary evaporator at 40 °C for 10 min to remove ethanol. The obtained RPH-SIF nanoparticles were stored in the dark or preserved by freeze drying. The nanoparticles prepared with RPH(A), RPH(N), RPH(T), and RPH(F) were named RPH(A)-SIF, RPH(N)-SIF, RPH(T)-SIF, and RPH(F)-SIF, respectively.

### 2.5. Characterization of RPH-SIF Nanoparticles 

#### 2.5.1. Determinations of Encapsulation Efficiency (EE) and Loading Capacity (LC) 

The EE and LC of RPH-SIF nanoparticles were measured using the method reported by Liu et al. [22] with slight modifications. In brief, 1 mL of RPH-SIF nanoparticle were added to 5 mL of ethyl acetate, vortexed for 30 s. The absorbance of the supernatant at 260 nm was measured using ethyl acetate as a blank control. The free flavonoid content was calculated according to the flavonoid standard curve equation. EE and LC were calculated as follows:

(1) $$EE\left(\%\right)=\frac{Total\;SIF\;content\left(mg\right)-Free\;SIF\;content\left(mg\right)}{Total\;SIF\;content\left(mg\right)}\times 100$$

(2) $$LC\left(\%\right)=\frac{Total\;SIF\;content\left(mg\right)-Free\;SIF\;content\left(mg\right)}{Total\;protein\;content\left(mg\right)}\times 100$$

#### 2.5.2. Measurements of Particle Size, Polydispersity Index (PDI), and Zeta Potential

The particle size, PDI, and zeta potential of the nanoparticles were obtained using a combined dynamic light scattering and particle electrophoresis instrument (90plus Zeta, Brookhaven Co., Ltd., Shanghai Branch, USA). The sample was diluted 10 times with deionized water, vortexed for 10 s, and then measured.

#### 2.5.3. Confocal Laser Scanning Microscopy (CLSM)

CLSM (Olympus FV3000, Tokyo, Japan) was performed to characterize the microstructure of RPH-SIF. RPHs were stained with Nile Blue (0.1%, *w/v*) dissolved in water [23], and SIF possess intrinsic fluorescence [24].

#### 2.5.4. Fourier Transform Infrared (FTIR) Spectroscopy 

RPHs and RPH-SIF nanoparticles, respectively, were mixed with potassium bromide at a ratio of 1:100 (RPHs/RPH-SIFs: potassium bromide, *w/w*). The samples were thoroughly ground until they were evenly mixed and then placed in an abrasive tablet tool to create uniformly transparent sheets. An FTIR spectrophotometer (NICOLET 380, Thermo Co., Ltd., Shanghai Branch, USA) was used to conduct full-wavelength (40~4000 cm^−1^) scanning analysis, where the resolution was set to 2 cm^−1^, and the scanning signal was accumulated 32 times.

#### 2.5.5. X-ray Diffraction (XRD)

The RPHs and RPH-SIF nanoparticles were weighed and then sealed in an aluminum foil bag. The XRD instrument (SMARTLAB9, Rigaku, Japan) was equipped with a copper K radiation source (λ = 0.154 nm). The detection conditions were as follows: nitrogen ventilation rate, 50 mL/min; heating rate, 10 °C/min; temperature setting range 20 °C to 260 °C.

#### 2.5.6. Fluorescence Spectroscopy

The fluorescence spectra of the RPH-SIFs were investigated using a fluorophotometer (F7100, Hitachi, Ltd., Tokyo, Japan). The final concentration of RPH was 1 mg/mL, and the concentration of encapsulated SIF was 0–60 μmol/L. RPH-SIF nanoparticles were determined by scanning emission wavelengths from 300 to 450 nm, with an excitation wavelength of 290 nm at 298, 304 and 310 K, to obtain the fluorescence spectra of the RPH-SIF nanoparticles. The excitation and emission slit widths were 5.0 nm.

The fluorescence quenching parameters were calculated from the following equations:

(3) $$\frac{F_{0}}{F}=1+K_{sv}[Q]$$

(4) $$\log\left(\frac{F_{0}-F}{F}\right)=logK_{A}-nlog\left[Q\right]$$

In Equation (3), F_0_ and F represent the fluorescence intensities of proteins in the absence and presence of SIF. K_sv_ represents the Stern–Volmer quenching constant, and [Q] represents the concentration of SIF. In Equation (4), K_A_ and n denote the binding constant and number of binding sites, respectively.

Equations (5) and (6) were used to calculate the thermodynamic parameters to determine the main driving force:

(5) $$lnK_{A}=-\frac{∆H}{RT}+\frac{∆S}{R}$$

(6) ∆G=∆H−T∆S

where ΔH and ΔS represent the enthalpy and entropy changes, respectively. R represents the gas constant (8.314 J·mol^−1^ K^−1^). T represents the thermodynamic temperature, and ΔG represents the change in Gibbs free energy. 

### 2.6. Antioxidant Activity of RPH-SIF Nanoparticles

#### 2.6.1. DPPH Radical Scavenging Activity 

The DPPH radical scavenging activity of the samples was determined based on the method reported by Xu et al. [14] with some modifications. Free SIF (in 70% ethanol) was diluted to a concentration of 5–40 μg/mL. The nanoparticles were diluted with deionized water to the same SIF concentration range (5–40 μg/mL). 2 mL of DPPH radical ethanol solution (100 μM) were mixed with 2 mL of samples and subsequently incubated at 25 °C in the dark for 30 min. The absorbance of the resulting solution was detected by a UV-Vis spectrophotometer (UV-1800, Shimadzu Corporation, Kyoto, Japan) at 517 nm. Deionized water was used as a blank control. The free radical scavenging activity of the different samples was calculated as follows:

(7) $$DPPH\;radical\;scavenging\;activity\left(\%\right)=\frac{Ac-As}{Ac}\times 100$$

where A_C_ and A_S_ represent the absorbance of the control and sample solutions, respectively.

#### 2.6.2. ABTS Radical Scavenging Activity

The ABTS radical scavenging activity of the samples was determined based on the method reported by Hu et al. [25] with some modifications. ABTS solution (7 mM) and potassium persulfate solution (2.45 mM) were mixed in equal volumes to create the working solution, which was then stored at 25 °C for 12–16 h in the dark. Phosphate buffer (pH 7.4, 5 mM) was used to dilute the ABTS working solution to an absorbance value of 0.70 ± 0.02. SIF in 70% ethanol was diluted to a concentration of 5–40 μg/mL, and the nanoparticles were diluted with deionized water to the same SIF concentration range. A 2.5-mL sample solution was mixed with 2.5 mL of ABTS solution and reacted at room temperature for 18 min. The absorbance of the reacted solution was measured by a UV-Vis spectrophotometer at 734 nm. Phosphate-buffered saline was used as a blank control. The ABTS radical scavenging activity of the samples was calculated using the following equation:

(8) $$ABTS\;radical\;scavenging\;activity\left(\%\right)=\frac{A_{C}-A_{S}}{A_{C}}\times 100$$

where A_C_ and A_S_ represent the absorbance of the control and sample solutions, respectively.

### 2.7. Stability of RPH-SIF Nanoparticles

#### 2.7.1. Thermal Stability

In order to explore the thermal stability, the solutions of RPH-SIF nanoparticles were heated at different temperature (60, 80 and 100 °C) in water bath for 30 min, and immediately cooled to room temperature (25 °C). The content of SIF was then measured using the method described in Section 2.5.2. The quantity of SIF that remained in the samples was calculated using the following equation:

(9) $$Retention\;rate\;of\;SIF\left(\%\right)=\frac{A_{0}-A_{S}}{A_{0}}\times 100$$

where A_0_ and A_S_ represent the absorbance of the unheated and heated sample solutions, respectively.

#### 2.7.2. Ionic Stability

Sodium chloride (NaCl) was added to the solutions of RPH-SIF nanoparticles to reach the final concentrations of NaCl at 0–250 mM. The particle size and PDI of the particles were determined as described in Section 2.5.2.

#### 2.7.3. pH Stability

The pH of the solutions of RPH-SIF nanoparticles were adjusted to 2–7 by 0.5 mol/L hydrochloric acid (HCl) and sodium hydroxide (NaOH). The particle size and PDI of the particles were determined as described in Section 2.5.2.

### 2.8. In Vitro Simulated Digestion

The in vitro simulated digestion model was constructed based on the method reported by Hu et al. [25,26] with some modifications. The simulated gastrointestinal tract model consisted of the gastric phase and intestinal phase, and the ingestion of RPH-SIF samples was monitored. Free SIF (pre-dissolved in ethanol) served as a control.

In the gastric phase, the solution of nanoparticles was mixed with pepsin (3.2 mg/mL), and the pH was adjusted to 2.5 with 0.1 mol/L HCl. The mixture was incubated at 37 °C for 2 h with continuous shaking. In the small intestine phase, trypsin was added to the sample solution. The mixture was adjusted to pH 7.4 with 1 mmol/L NaOH and incubated at 37 °C for 6 h with continuous shaking. The sample was maintained at 4 °C to inactivate the enzyme, and the SIF content of the samples were measured every 30 min, using the method described in Section 2.5.1. The quantity of SIF remaining in the samples was calculated using the following equation:

(10) $$Retention\;rate\;of\;SIF\left(\%\right)=\frac{A_{S}-A_{0}}{A_{0}}\times 100$$

where A_0_ and A_S_ represent the absorbance of the sample solutions at 0 min and other times (min), respectively.

### 2.9. Statistical Analysis 

All experiments were conducted three times, and the data were presented as the mean ± standard deviation. One-way ANOVA with Duncan’s multiple range test was used to analyze the data and determine significant differences among samples. Significance analysis tests were conducted in IBM SPSS v.27. *p* values were considered statistically significant at <0.05.

## 3. Results and Discussion

### 3.1. Effects of the DH and Molecular Weight of RPH on the Fabrication of Nanoparticles

**Figure 1:**
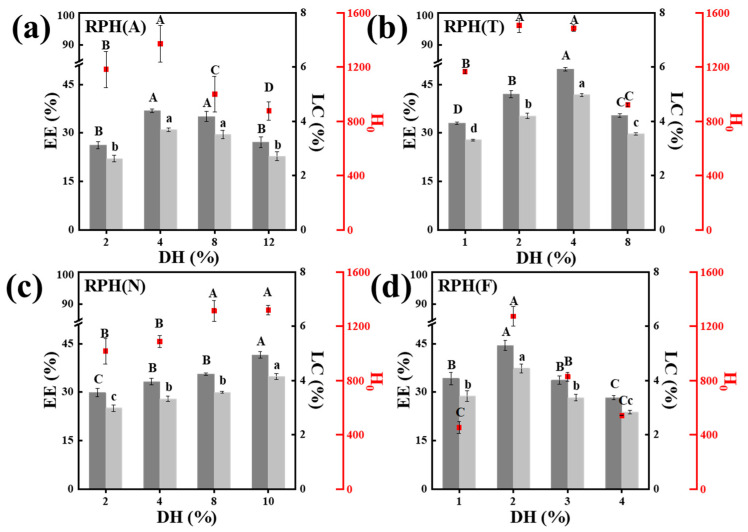
The encapsulation efficiency (EE) and loading capacity (LC) of rice protein hydrolysate soy isoflavone (RPH-SIF) nanoparticles at different degrees of hydrolysis (DH) of RPHs ((**a**): RPH(A), (**b**): RPH(T), (**c**): RPH(N), and (**d**): RPH(F)), the hydrophobicity (H_0_) of RPHs with different DHs. ^A–D^ Different superscript letters represent a significant difference (*p* < 0.01). ^a–d^ Different superscript letters represent a significant difference (*p* < 0.05).

As shown in Figure 1a–d, the EE and LC of nanoparticles produced by RPHs of different DHs generally presented a trend of first increasing and then decreasing. When the highest EE and LC were obtained, the DHs of the RPHs were different according to the protease. The optimum DHs of RPs hydrolyzed by different proteases were 4%, 4%, 10% and 2% for alcalase, neutrase, trypsin, and flavorzyme, respectively. With the increase in DH, the H_0_ of RPHs also changed as similar as the corresponding EE and LC. Moderate hydrolysis leads to the exposure of hydrophobic groups, and then further enzymatic hydrolysis may destroy the hydrophobic areas [27], which resulted in the decrease of H_0_. Usually, the H_0_ of a protein can be used as an indicator of the number of hydrophobic groups bound to the polar solution environment [19]. Consequently, the H_0_ can affect the efficacy of the complexation of SIF with RPHs by influencing the amphiphilic nature of RPHs [28].

**Figure 2:**
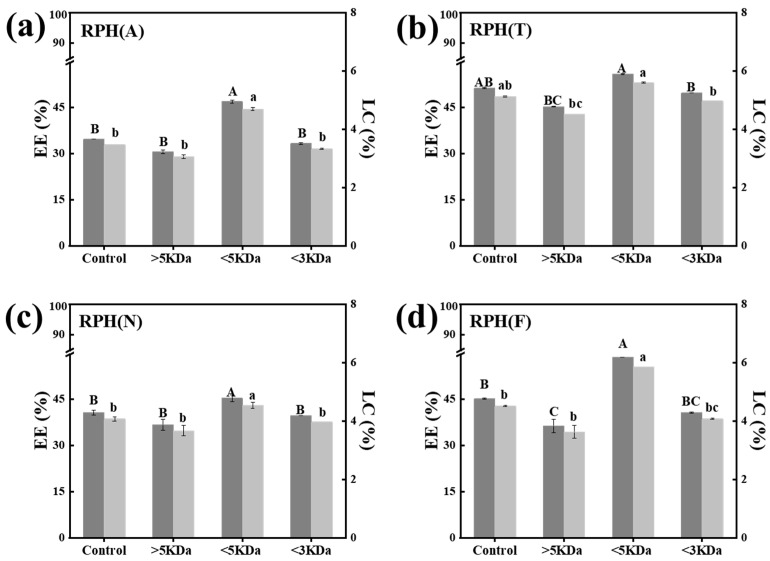
The encapsulation efficiency (EE) and loading capacity (LC) of rice protein hydrolysate soy isoflavone (RPH-SIF) nanoparticles with different molecular weights of RPH ((**a**): RPH(A), (**b**): RPH(T), (**c**): RPH(N), and (**d**): RPH(F)), ^A–C^ Different superscript letters represent a significant difference (*p* < 0.01). ^a–c^ Different superscript letters represent a significant difference (*p* < 0.05).

Figure 2a–d show that the highest EE and LC of nanoparticles were obtained when the molecular weight of the RPHs was <5 kDa. The molecular weight distribution will affect the packaging of nanoparticles [29]. The protein was hydrolyzed into shorter peptides, thus the functional properties changed [30,31]. The high EE and LC may be attributed to the moderate amphiphilicity and structure of the RPHs, which contributed to the encapsulation of SIF by peptide self-assembly [32]. 

### 3.2. Optimization of the Preparation Conditions of RPH-SIF Nanoparticles

The influence of temperature, time, pH, and the mass ratio between RPH and SIF on the formation of nanoparticles are shown in Table S2, while the optimized preparation conditions for RPH-SIF nanoparticles are listed in Table 1. The smaller particle size, lowest zeta potential and PDI indicated a more stable nano-system [33]. Hence, the best preparation conditions of RPH-SIF nanoparticles were selected according to higher EE and LC, and the better results of DSL. Among the RPH-SIFs, RPH(F)-SIF had the highest EE (90.65%) and LC (9.07%), the smallest particle size (64.77 nm), the lowest PDI (0.19), and the lowest zeta potential (−25.64 mV). These results indicated that RPH(F) had a higher packed load capacity for SIF at the same RPH/SIF mass ratio compared with the other RPHs, and that RPH(F)-SIF was more stable than the nanoparticles that were synthesized with the other proteases.

**Figure 3:**
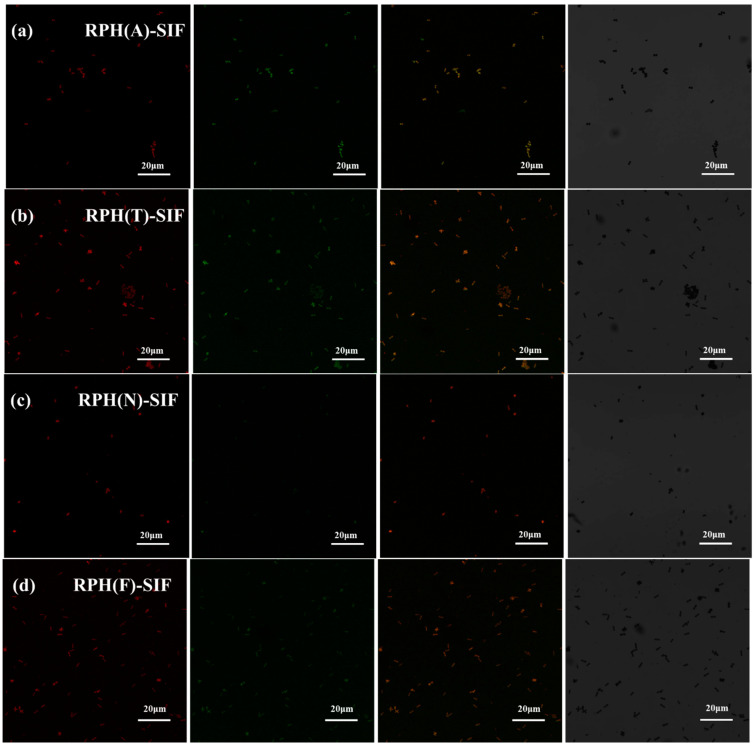
The CLSM images of RPH(A)-SIF (**a**), RPH(T)-SIF (**b**), RPH(N)-SIF (**c**), and RPH(F)-SIF (**d**). Red and green represented RPHs and SIF, respectively.

CLSM is used to further characterize the microstructure of nanoparticles. As shown in Figure 3, RPHs was red by stained with Nile Blue (the first column), and SIF was green for its inherent fluorescence (the second column). The pictures in the third column were the superpositions of the two pictures on the left, indicating the successful encapsulation, which is consistent with the results of FTIR (Figure 3a) and XRD (Figure 3b). The fourth column was the bright field images of different RPH-SIF nanoparticles. The EE of RPH(N)-SIF is low, so it is not obvious after stacking. And the EE of RPH(T)-SIF and RPH(F)-SIF is relatively high, but the dispersion coefficient of RPH(F)-SIF is the lowest. The results could be consistent with the results of DLS.

### 3.3. Interaction of RPH-SIF Nanoparticles

#### 3.3.1. FTIR and XRD Analysis

**Figure 4:**
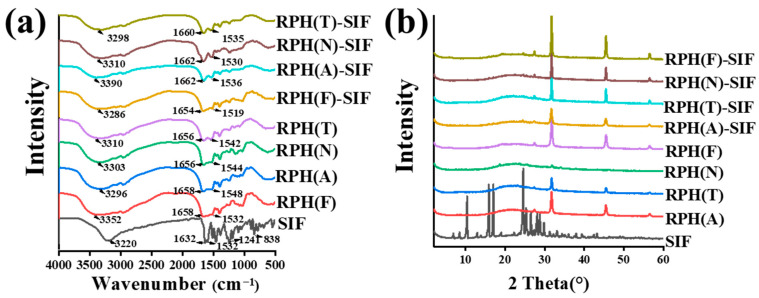
Fourier transform infrared spectra (**a**) and X-ray diffraction (XRD) patterns (**b**) of SIF, RPH, and RPH-SIF complex nanoparticles.

The interactions between molecules in the nanoparticles were characterized using FTIR spectroscopy (Figure 4a). The characteristic bands of SIF at 1241 cm^−1^ and 838 cm^−1^ did not appear in the spectra of RPH-SIF nanoparticles. This result demonstrated that SIF was successfully entrapped within the nanoparticles. A broad band between 3100 and 3500 cm^−1^ appeared in all the spectra, which corresponded to the O–H stretching vibration [34]. In the RPH(F), RPH(A), RPH(N), and RPH(T) spectra, characteristic bands of O–H appeared at 3352 cm^−1^, 3296 cm^−1^, 3303 cm^−1^, and 3310 cm^−1^, respectively. However, the O–H bands changed to 3286 cm^−1^, 3390 cm^−1^, 3310 cm^−1^, and 3298 cm^−1^ in the RPH(F)-SIF, RPH(A)-SIF, RPH(N)-SIF, and RPH(T)-SIF spectra, respectively. And the phenolic hydroxyl groups were involved in the non-covalent interaction between RPH and SIF [35]. After the formation of nanoparticles, the various shifts of O–H band observed may be related to the secondary structure of the RPHs [36]. In the RPH spectra, there were two obvious bands at around 1658 cm^−1^ and 1542 cm^−1^, which represent the amide I (C=O stretching vibration) and amide II band (N=H in-plane bending, C–C stretching vibration, and C–N stretching vibration), respectively [23]. The bands of the amide I band changed to 1654 cm^−1^, 1662 cm^−1^, 1662 cm^−1^, and 1660 cm^−1^ for RPH(F)-SIF, RPH(A)-SIF, RPH(N)-SIF, and RPH(T)-SIF, respectively, while the bands of the amide II band changed to 1519 cm^−1^, 1536 cm^−1^, 1530 cm^−1^, and 1535 cm^−1^, respectively. There is a slight shift in complex nanoparticles, which confirms the electrostatic interactions between RPH and SIF [37]. These results demonstrate that hydrogen bonds and electrostatic interactions exist between RPH and SIF during the formation of complex nanoparticles. 

The XRD patterns of the SIF, RPH, and RPH-SIF nanoparticles are shown in Figure 4b. The spectra of RPHs were smooth curves, indicating that they were in an amorphous state. Some peaks at the 2θ of 31.74° and 45.50° were attributed to the presence of NaCl formed as a result of using NaOH and HCl to adjust the pH [38]. The SIF had sharp characteristic peaks, indicating that it had high crystallinity. However, when encapsulated in nanoparticles, the XRD peaks of SIF disappeared, indicating that SIF had been successfully encapsulated in the nanoparticles in an amorphous form. It has been reported previously that the XRD patterns of Cur, RPs, and RPCNs3 showed similar patterns, where the characteristic peaks of flavonoids disappeared after encapsulation [14].

#### 3.3.2. Fluorescence Spectroscopy Analysis

The intrinsic fluorescence of proteins is a valuable property for obtaining local information about the conformational and/or dynamic changes of proteins binding with small molecules in aqueous solutions [14]. The intrinsic fluorescence of RPH influenced by SIF was measured to study the binding mechanism of SIF to RPH. After the addition of SIF, the fluorescence of RPH was gradually quenched with increasing SIF concentration (Figure S2). This result illustrated that strong interactions existed between RPH and SIF [39]. The Stern–Volmer equation was applied to characterize the interaction between RPH and SIF, and the results are listed in Table 2. The K_sv_ values of all RPH-SIF nanoparticles showed a decreasing trend with increasing temperature, which showed that during the formation of the nanoparticles, the quenching of RPH by SIF is a static quenching phenomenon. Static quenching refers to the formation of a complex between a quencher and a fluorophore due to the coordination reaction [40]. The relative values of enthalpy (ΔH) to entropy (ΔS) can help to determine the major forces involved in the interaction between a ligand and proteins [41]. As shown in Table 2, the interaction between RPH and SIF yielded negative ΔH and ΔS values, implying that the binding was driven by hydrogen bonding and electrostatic interaction attraction [41], which was in accordance with the FTIR spectral data. The Gibbs free energy (ΔG) value (<0) indicated that the reaction was spontaneous [19]. The value of n indicated the number of binding sites between RPH and SIF. RPH(F)-SIF had the largest binding site constant among the RPH-SIF nanoparticles, reaching 1 at 298 K, which demonstrated that RPH(F) had the strongest binding with SIF [19]. This result was consistent with the result that the highest EE and LC were achieved by the RPH(F)-SIF nanoparticles.

#### 3.3.3. Amino Acid Profile Analysis

The amino acid compositions (g/100 g of protein) of RP and the RPHs at the optimal DH are shown in Table S3. The differences in the amino acid compositions between the RPHs were mainly attributed to the differences in the specificity of the enzyme used. After enzymatic hydrolysis, the amino acid composition of negatively charged amino acids in RPH increased significantly, and the total amount of hydrophilic amino acids (HAAs) increased for all RPHs. The amount of HAAs in RPH(F) was the highest, resulting in a better interaction between RPH(F) and SIF through hydrogen bonds [42].

### 3.4. Evaluation of the Stability of RPH-SIF Nanoparticles

#### 3.4.1. Thermal Stability

**Figure 5:**
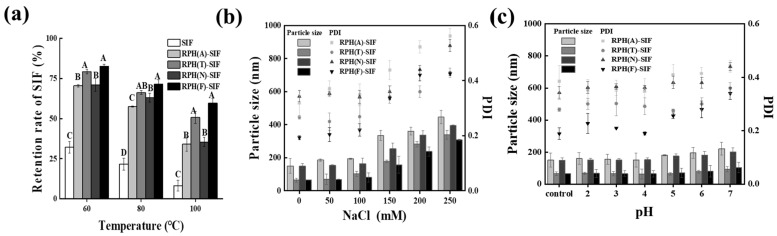
The effects of temperature (**a**) on the retention of SIF in nanoparticles and the effects of ionic strength (**b**) and pH (**c**) on the particle size and PDI of SIF-loaded RPHs. ^A–D^ Different superscript letters represent a significant difference (*p* < 0.05).

As shown in Figure 5a, after thermal treatment at 60 °C, 80 °C and 100 °C, the SIF contents in the free SIF solution were reduced to 32.11%, 21.62% and 8.23%, respectively. In comparison, the retention of SIF in nanoparticles was significantly improved. RPH(F)-SIF had the best thermal stability compared to the other RPH-SIF nanoparticles. The contents of SIF in RPH(F)-SIF after thermal treatment at 60 °C, 80 °C and 100 °C were 82.65%, 71.47% and 59.67%, respectively, and were enhanced 2.57-fold, 3.31-fold, and 7.25-fold, respectively, compared with free SIF at the same temperatures. The better thermal stability of RPH(F)-SIF and RPH(T)-SIF may be due to that the preparation of corresponding RPHs underwent a higher temperature, and then the nanoparticles obtained a higher heat resistance. After the formation of nanoparticles, the stability of SIF in the system was improved due to the interaction between RPHs and SIF. These phenomena indicated that the RPH nanoparticles have a protective effect on SIF under heat treatment. 

#### 3.4.2. Ionic Stability

The influence of ionic concentration on the particle size and PDI of complex nanoparticles is presented in Figure 5b. When the concentration of NaCl was 0 to 100 mM, the nanoparticles showed excellent stability, and the particle size and PDI did not change significantly. As the NaCl concentration rose to 150 mM, all RPH-SIF nanoparticles aggregated and their particle size grew. In the low concentration salt solution, RPH(F)-SIF showed the smallest particle size and the lowest PDI, indicating that it had the best stability at low ionic concentrations. The extent of precipitation was enhanced with the further increase in NaCl concentration because the presence of NaCl results in electrostatic shielding of complex nanoparticles. Specifically, excess Na^+^ and Cl^−^ neutralized the charge of nanoparticles, resulting in reduced electrostatic repulsion [43].

#### 3.4.3. pH Stability

The influence of pH on the particle size and PDI of the complex nanoparticles is presented in Figure 5c. Nanoparticles show better pH stability in acidic environments. The particle size and PDI of the nanoparticles gradually increased with rising pH, and aggregation had already started to take place in a neutral environment. The pH of the environment in the human body is 2–7. In the alkaline environment, the interaction between nanoparticles was destroyed, and the structure of the hydrolysate was changed, leading to aggregation and sedimentation [44]. Due to the different capacity of interactions between RPH and SIF, they exhibit different pH stability capabilities [16], which corresponds to the results of the fluorescence interaction analysis. Therefore, it is difficult to form stable nanoparticles between RPH and SIF in an alkaline environment.

### 3.5. Antioxidant Activity of RPH-SIF Nanoparticles

**Figure 6:**
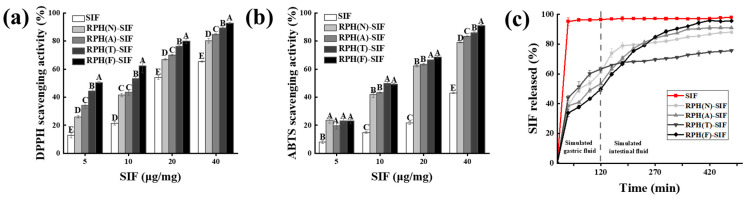
DPPH (**a**) and ABTS scavenging activities (**b**) of free and encapsulated SIF. (**c**) Release percentage of SIF in RPHs during in vitro digestion. ^A–E^ Different superscript letters represent a significant difference (*p* < 0.05).

It has been reported that SIF can protect the body from excessive reactive oxygen species and free radicals and reduce the damage of various chronic diseases [25]. In the current study, the determination of antioxidant activity was carried out using DPPH and ABTS assays. Figure 6a,b show that the DPPH and ABTS scavenging activities of nanoparticles were significantly higher than those of free SIF. Furthermore, the antioxidant activity of RPH(F)-SIF was significantly higher than that of the other nanoparticles (*p* < 0.05). The DPPH and ABTS scavenging activities of RPH(F)-SIF were increased by 27.36 and 48.01%, respectively, compared to that of free SIF. The improved oxidation resistance of the nano-system and the water dispersibility of SIF may have contributed to the tight binding of RPH-SIF nanoparticles, which provided strong steric repulsion and hydrophilicity and facilitated the reaction with free radicals in the aqueous phase [22].

### 3.6. In Vitro Release of RPH-SIF Nanoparticles

The release behavior of SIF is presented in Figure 6c. The release rate of SIF encapsulated in nanoparticles was visually slowed. In addition, RPH(F)-SIF exhibited a less release of SIF throughout the simulated gastric juice period compared to the other RPH-SIF nanoparticles. First, at the end of the gastric-release phase, the amount of SIF released from the nanoparticles was 61%, 53%, 63% and 49% for RPH(N)-SIF, RPH(A)-SIF, RPH(T)-SIF, and RPH(F)-SIF, respectively. The amounts of SIF released after 8 h of incubation were 88%, 91%, 75% and 96% for RPH(N)-SIF, RPH(A)-SIF, RPH(T)-SIF, and RPH(F)-SIF, respectively. The results show that a rapid release of SIF occurred in the beginning of gastric-release phase. This is due to the entry of nanoparticles into a highly acidic environment, cou-pled with the action of pepsin and the role of high salt ions, which destroys the structure of nanoparticles, leading to the extremely rapid release of SIF. And The results show that RPH has an obvious slow-releasing effect on SIF, which may be due to the increased resistance of RPH to trypsin and pepsin and the weak interaction between RPH and SIF. Moreover, the release characteristics of different nanoparticles were related the corresponding RPH. The influences of RPHs may be due to their different structure obtained by the hydrolysis of different enzymes, which was further affected the interaction between RPH and SIF. Because the absorption site of SIF is in the intestinal epithelial cells [35], the RPH-SIF needed to ensure sufficient release of SIF in the intestine. These results also suggested that a large amount of SIF from the RPH(F)-SIF delivery system will reach the colon, where it can exert a beneficial effect [45]. Therefore, RPH(F)-SIF is an effective SIF delivery system for the release of SIF.

## 4. Conclusions

In this study, SIF and RPHs were manufactured into bioactive compound-loaded nanoparticles using the anti-solvent method. The nanoparticles are stabilized by hydrogen bonds and work together with other interactions, such as hydrophobic interactions and electrostatic interactions. In the comparison of RPH-SIF nanoparticles prepared by different enzymatic hydrolysates. In the comparison of RPH-SIF nanoparticles prepared by different enzymatic hydrolysates, RPH(F)-SIF had the highest EE (90.67%) and LC (9.06%), the smallest particle size (64.77 nm), the lowest PDI (0.19), and the lowest zeta potential (−25.64 mV). Better stability, anti-oxidant properties, and targeted releasing effect for SIF were also obtained in RPH(F)-SIF. These findings demonstrate a novel method for the use of proteins as natural carriers for hydrophobic bioactive substance delivery.

## Figures and Tables

**Table 1:** Optimization of the preparation conditions of RPH-SIF nanoparticles.

	RPH(A)-SIF	RPH(T)-SIF	RPH(N)-SIF	RPH(F)-SIF
Temperature (°C)	40	55	40	55
Time (min)	60	60	60	60
pH	5	5	3	4
SIF/RPH ratios	1/10	1/10	1/10	1/10
EE (%)	61.16 ± 0.92	82.86 ± 1.32	77.62 ± 0.31	90.65 ± 0.19
LC (%)	6.12 ± 0.09	8.29 ± 0.13	7.62 ± 0.03	9.06 ± 0.02
Particle size (nm)	77.61 ± 28.31	64.84 ± 11.53	92.91 ± 4.53	64.77 ± 1.34
Zeta potential (mV)	−21.35 ± 1.03	−19.45 ± 0.88	−20.45 ± 3.24	−25.64 ± 0.63
PDI	0.3 ± 0.02	0.27 ± 0.01	0.34 ± 0.02	0.19 ± 0.02

**Table 2:** Relevant parameters of the Stern-Volmer quenching constant and the values of thermodynamic parameters.

Parameters	RPH(A)-SIF			RPH(T)-SIF			RPH(N)-SIF			RPH(F)-SIF		
	298 K	304 K	310 K	298 K	304 K	310 K	298 K	304 K	310 K	298 K	304 K	310 K
K_SV_ (L/mol)	8802	7599	7122.5	15,288	7473.5	6586.6	10,082	10,370	5994.7	9191.4	8348.3	4170.2
K_A_ × 10^6^ (L/mol)	1.63	1.36	1.23	2.79	2.54	2.23	2.04	1.99	1.17	4.09	3.54	2.61
n	0.42	0.37	0.34	0.66	0.68	0.60	0.51	0.59	0.34	1.01	0.89	0.70
△H (KJ/mol)	−18			−14.34			−35.12			−28.79		
△S (J/mol)	−56.42			−39.51			−110.78			−84.66		
△G (KJ/mol)	−1.18	−0.84	−0.51	−2.56	−2.32	−2.09	−2.11	−1.44	−0.78	−3.57	−3.06	−2.55

## Data Availability

Data is contained within the article or supplementary material.

## Supplementary Materials

The following supporting information can be download at: https://www.mdpi.com/article/10.3390/foods12071523/s1. Figure S1. The curves of the degree of hydrolysis (a) and molecular weight distribution (b) of RPHs by different enzymes; Figure S2. The intrinsic fluorescence quenching of RPH (a: RPH(A), b: RPH(N), c: RPH(T), and d: RPH(F)) by SIF at 298 K, 304 K, and 310 K. The concentration of SIF was 0–60 μmol/L; Table S1. Hydrolysis conditions of different proteases; Table S2. Optimization of the preparation conditions of RPH-SIF nanoparticles; Table S3. Amino acid compositions (g/100 g of protein) of RP and RPHs.
