# Supplementary material for: Formation and Characterization of Self-Assembled Rice Protein Hydrolysate Nanoparticles as Soy Isoflavone Delivery Systems
Source: Foods. 2023 Apr 4;12(7):1523. doi: 10.3390/foods12071523 (PMC10094372; doi:10.3390/foods12071523)
Supplement: Supplementary file 1 [file foods-12-01523-s001.zip › foods-2291939-supplementary.docx]

**Supplementary material**

**Figure. S1** The curves of the degree of hydrolysis (a) and molecular weight distribution (b) of RPHs by different enzymes.

**Figure. S2** The intrinsic fluorescence quenching of RPH (a: RPH(A), b: RPH(N), c: RPH(T), and d: RPH(F)) by SIF at 298 K, 304 K, and 310 K. The concentration of SIF was 0–60 μmol/L.

**Table. S1** Hydrolysis conditions of different proteases.

**Table. S2** Optimization of the preparation conditions of RPH-SIF nanoparticles.

**Table. S3** Amino acid compositions (g/100 g of protein) of RP and RPHs.


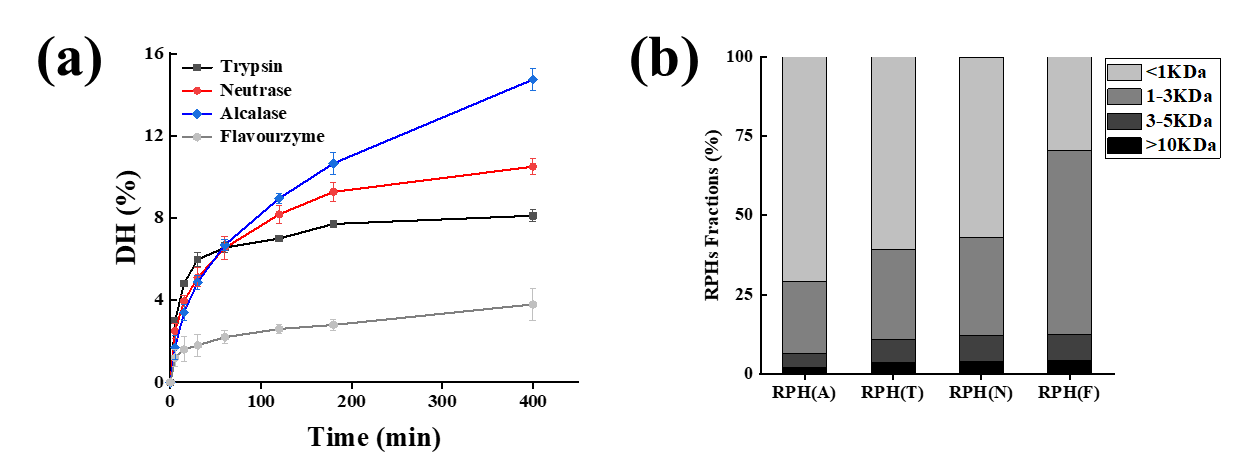
Figure. S1 The curves of the degree of hydrolysis (a) and molecular weight distribution (b) of RPHs by different enzymes.

Figure. S2 The intrinsic fluorescence quenching of RPH (a: RPH(A), b: RPH(N), c: RPH(T), and d: RPH(F)) by SIF at 298 K, 304 K, and 310 K. The concentration of SIF was 0–60 μmol/L.


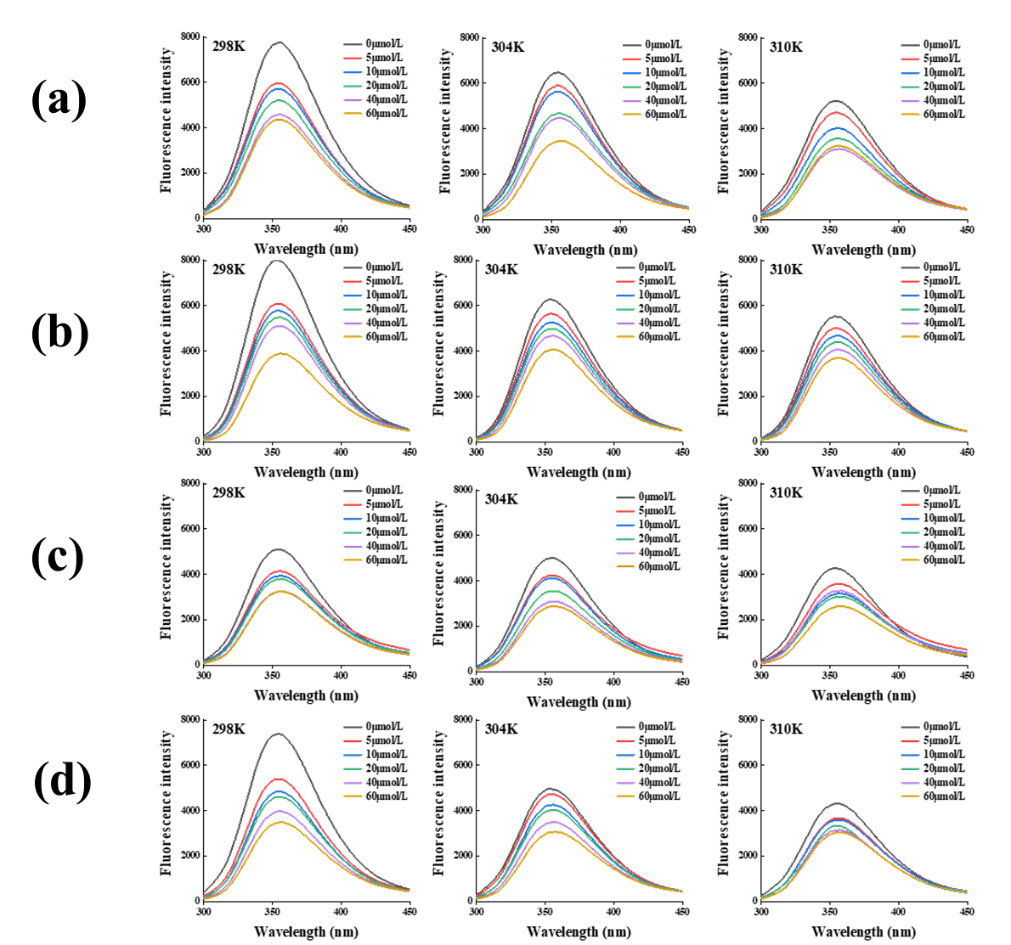


Table. S1 Hydrolysis conditions of different proteases.

|  | **Alcalase** | **Neutrase** | **Trypsin** | **Flavourzyme** |
| --- | --- | --- | --- | --- |
| Activity(U/mg) | 212.32 | 217.43 | 52.19 | 33.68 |
| Main enzymolysis sites | Broad specificity | Arg-His | Broad specificity | Leu-Pro, Pro-Pro |
| Temperature (℃) | 45 | 50 | 45 | 50 |
| pH | 9 | 8 | 7 | 6 |
| Enzyme/RP ratio (w/w) | 1/100 | 1/100 | 1/25 | 1/15 |

Table. S2 Optimization of the preparation conditions of RPH-SIF nanoparticles.

| **Temp (℃)** | **Time (min)** | **SIF/RPH ratios** | **pH** | **EE (%)** | **LC (%)** | **Particle size (nm)** | **Zeta potential (mv)** | **PDI** |
| --- | --- | --- | --- | --- | --- | --- | --- | --- |
| **RPH(A)-SIF** | | | | | | | | |
| 25 | 60 | 1/10 | 7 | 49.44±2.22 | 4.94±0.23 | 140.80±20.97 | -21.73±0.85 | 0.37±0.02 |
| 40 |  |  |  | 53.63±0.28 | 5.36±0.03 | 111.60±21.93 | -21.29±0.56 | 0.34±0.01 |
| 55 |  |  |  | 40.28±1.77 | 4.03±0.18 | 228.32±25.52 | -18.03±1.16 | 0.39±0.02 |
| 70 |  |  |  | 36.04±1.71 | 3.60±0.17 | 255.93±48.91 | -18.58±2.39 | 0.36±0.01 |
| 40 | 10 | 1/10 | 7 | 19.25±1.25 | 1.93±0.13 | 234.79±11.28 | -21.79±2.22 | 0.32±0.02 |
|  | 30 |  |  | 25.43±1.47 | 2.54±0.15 | 285.32±14.68 | -21.68±0.02 | 0.34±0.02 |
|  | 60 |  |  | 53.61±0.74 | 5.36±0.07 | 95.79±1.28 | -21.21±0.32 | 0.34±0.01 |
|  | 90 |  |  | 50.25±2.64 | 5.03±0.26 | 99.36±2.36 | -13.45±2.05 | 0.33±0.03 |
| 40 | 60 | 1/5 | 7 | 36.61±1.25 | 7.40±0.13 | 94.79±5.45 | -19.09±4.48 | 0.34±0.02 |
|  |  | 1/10 |  | 53.45±3.27 | 5.35±0.33 | 96.85±6.54 | -21.90±5.01 | 0.34±0.02 |
|  |  | 1/20 |  | 55.85±2.57 | 2.79±0.02 | 134.79±6.28 | -21.79±2.22 | 0.41±0.01 |
|  |  | 1/40 |  | 20.05±1.64 | 0.50±0.11 | 185.32±6.68 | -20.68±0.02 | 0.46±0.02 |
| 40 | 60 | 1/10 | 3 | 59.51±0.25 | 5.95±0.03 | 408.57±3.60 | -16.17±3.60 | 0.33±0.02 |
|  |  |  | 4 | 63.68±0.67 | 6.37±0.07 | 167.12±4.75 | -20.46±2.28 | 0.36±0.01 |
|  |  |  | 5 | 61.16±0.92 | 6.12±0.09 | 77.61±28.31 | -21.35±1.03 | 0.30±0.02 |
|  |  |  | 6 | 54.73±2.20 | 5.47±0.22 | 158.63±9.85 | -20.81±5.01 | 0.26±0.02 |
|  |  |  | 7 | 50.62±2.82 | 5.06±0.28 | 136.77±20.39 | -23.79±2.22 | 0.44±0.01 |
|  |  |  | 8 | 27.79±1.73 | 2.78±0.17 | 348.21±13.81 | -25.68±0.02 | 0.32±0.02 |
|  |  |  | 9 | 26.29±2.09 | 2.62±0.21 | 343.38±22.61 | -33.25±0.32 | 0.41±0.01 |
| **RPH(T)-SIF** | | | | | | | | |
| 25 | 60 | 1/10 | 7 | 40.34±0.58 | 4.03±0.06 | 117.38±24.20 | -18.90±1.63 | 0.34±0.03 |
| 40 |  |  |  | 52.33±0.92 | 5.23±0.09 | 74.08±1.72 | -19.56±1.82 | 0.35±0.02 |
| 55 |  |  |  | 66.89±2.08 | 6.69±0.21 | 51.85±3.98 | -17.77±2.17 | 0.34±0.04 |
| 70 |  |  |  | 16.16±3.39 | 1.62±0.34 | 285.49±60.70 | -18.97±2.59 | 0.37±0.02 |
| 55 | 10 | 1/10 | 7 | 42.35±0.93 | 4.24±0.09 | 52.97±20.38 | -22.30±1.95 | 0.36±0.03 |
|  | 30 |  |  | 45.77±2.40 | 4.58±0.24 | 74.08±1.72 | -20.59±0.80 | 0.35±0.02 |
|  | 60 |  |  | 61.47±0.98 | 6.15±0.10 | 53.85±3.98 | -17.65±2.17 | 0.34±0.04 |
|  | 90 |  |  | 43.91±0.33 | 4.39±0.03 | 286.49±60.70 | -18.98±2.09 | 0.29±0.04 |
| 55 | 60 | 1/5 | 7 | 47.12±2.87 | 9.42±0.57 | 62.79±3.24 | -19.09±4.48 | 0.34±0.02 |
|  |  | 1/10 |  | 61.47±0.98 | 6.15±0.10 | 54.52±2.26 | -20.90±5.01 | 0.32±0.02 |
|  |  | 1/20 |  | 56.72±1.52 | 2.84±0.08 | 202.79±1.23 | -20.79±2.22 | 0.34±0.01 |
|  |  | 1/40 |  | 43.23±1.06 | 1.08±0.27 | 205.32±4.68 | -20.68±0.02 | 0.33±0.02 |
| 55 | 60 | 1/10 | 3 | 74.02±0.52 | 7.40±0.05 | 148.81±14.53 | -13.27±2.90 | 0.40±0.04 |
|  |  |  | 4 | 80.21±0.31 | 8.02±0.03 | 162.14±36.62 | -18.94±1.61 | 0.25±0.03 |
|  |  |  | 5 | 82.86±1.32 | 8.29±0.13 | 64.86±11.53 | -19.45±0.88 | 0.27±0.01 |
|  |  |  | 6 | 50.96±0.93 | 5.96±0.09 | 78.29±8.31 | -19.66±2.21 | 0.34±0.02 |
|  |  |  | 7 | 35.27±3.28 | 3.53±0.32 | 181.72±15.75 | -20.76±0.51 | 0.36±0.02 |
|  |  |  | 8 | 28.70±4.81 | 2.87±0.48 | 286.49±60.68 | -22.27±0.02 | 0.35±0.02 |
|  |  |  | 9 | 25.90±2.26 | 2.59±0.23 | 33.16±2.24 | -26.03±1.61 | 0.44±0.01 |
| **RPH(N)-SIF** | | | | | | | | |
| 25 | 60 | 1/10 | 7 | 41.67±2.11 | 4.17±0.21 | 69.97±1.93 | -23.50±2.83 | 0.35±0.01 |
| 40 |  |  |  | 58.70±1.03 | 5.87±0.10 | 95.15±2.39 | -25.53±1.11 | 0.36±0.06 |
| 55 |  |  |  | 43.27±2.57 | 4.33±0.26 | 100.70±10.04 | -19.97±1.07 | 0.34±0.01 |
| 70 |  |  |  | 20.16±0.93 | 2.02±0.09 | 69.16±18.84 | -23.03±1.61 | 0.41±0.05 |
| 40 | 10 | 1/10 | 7 | 18.77±1.97 | 1.88±0.20 | 91.85±6.45 | -21.20±0.65 | 0.34±0.01 |
|  | 30 |  |  | 28.80±1.19 | 2.88±0.12 | 92.79±2.53 | -22.13±1.14 | 0.33±0.01 |
|  | 60 |  |  | 58.97±3.67 | 5.90±0.37 | 95.17±3.04 | -25.68±1.18 | 0.34±0.01 |
|  | 90 |  |  | 51.47±1.12 | 5.15±0.11 | 70.98±35.45 | -16.25±1.62 | 0.40±0.07 |
| 40 | 60 | 1/5 | 7 | 36.76±2.58 | 7.34±1.15 | 100.85±3.26 | -22.45±3.24 | 0.36±0.01 |
|  |  | 1/10 |  | 58.85±2.64 | 5.89±0.26 | 95.79±1.28 | -25.13±2.55 | 0.34±0.01 |
|  |  | 1/20 |  | 51.45±3.54 | 2.59±0.07 | 130.36±12.36 | -21.68±1.05 | 0.40±0.01 |
|  |  | 1/40 |  | 25.45±3.83 | 0.63±0.10 | 170.98±14.45 | -19.25±0.54 | 0.47±0.07 |
| 40 | 60 | 1/10 | 3 | 77.62±0.31 | 7.76±0.03 | 92.91±4.53 | -20.45±3.24 | 0.34±0.02 |
|  |  |  | 4 | 73.95±1.74 | 7.40±0.17 | 95.79±1.28 | -20.35±0.32 | 0.26±0.01 |
|  |  |  | 5 | 61.02±2.01 | 6.10±0.20 | 99.36±2.36 | -20.98±2.05 | 0.31±0.01 |
|  |  |  | 6 | 52.51±1.71 | 5.25±0.17 | 92.79±5.45 | -21.56±4.48 | 0.34±0.02 |
|  |  |  | 7 | 39.70±3.83 | 3.97±0.38 | 96.85±7.26 | -21.90±5.01 | 0.36±0.02 |
|  |  |  | 8 | 16.80±4.98 | 1.68±0.50 | 234.79±11.28 | -21.79±2.22 | 0.44±0.01 |
|  |  |  | 9 | 5.91±1.94 | 0.60±0.20 | 285.32±14.68 | -23.68±0.02 | 0.43±0.02 |
| **RPH(F)-SIF** | | | | | | | | |
| 25 | 60 | 1/10 | 7 | 24.90±11.07 | 2.49±1.11 | 80.19±9.09 | -28.69±1.65 | 0.34±0.00 |
| 40 |  |  |  | 74.92±0.84 | 7.49±0.08 | 100.25±9.12 | -23.99±0.54 | 0.36±0.02 |
| 55 |  |  |  | 78.72±0.85 | 7.87±0.09 | 45.80±5.21 | -18.25±1.19 | 0.30±0.09 |
| 70 |  |  |  | 45.67±0.87 | 4.57±0.09 | 120.47±17.10 | -20.17±0.85 | 0.39±0.02 |
| 55 | 10 | 1/10 | 7 | 45.80±1.01 | 4.58±0.10 | 74.81±0.48 | -18.52±1.06 | 0.48±0.12 |
|  | 30 |  |  | 48.26±0.67 | 4.83±0.07 | 96.82±3.89 | -20.38±1.13 | 0.46±0.06 |
|  | 60 |  |  | 78.45±0.67 | 7.85±0.07 | 46.25±1.68 | -18.25±1.19 | 0.31±0.04 |
|  | 90 |  |  | 46.69±2.41 | 4.67±0.24 | 173.18±10.15 | -21.15±0.62 | 0.38±0.02 |
| 55 | 60 | 1/5 | 7 | 53.87±1.73 | 10.77±0.35 | 65.25±3.68 | -16.09±2.05 | 0.45±0.02 |
|  |  | 1/10 |  | 78.89±1.24 | 7.89±0.12 | 45.87±1.11 | -19.04±0.54 | 0.31±0.01 |
|  |  | 1/20 |  | 54.07±1.71 | 2.71±0.08 | 98.75±8.28 | -20.79±2.21 | 0.34±0.01 |
|  |  | 1/40 |  | 50.65±3.91 | 1.27±0.10 | 102.34±6.68 | -21.64±0.38 | 0.41±0.02 |
| 55 | 60 | 1/10 | 3 | 67.29±4.56 | 6.73±0.45 | 164.10±15.51 | -20.35±2.08 | 0.29±0.02 |
|  |  |  | 4 | 90.65±0.19 | 9.06±0.02 | 64.77±1.34 | -25.64±0.63 | 0.19±0.02 |
|  |  |  | 5 | 80.89±0.78 | 8.09±0.08 | 74.32±11.15 | -26.45±0.97 | 0.32±0.02 |
|  |  |  | 6 | 62.44±3.53 | 6.24±0.35 | 75.63±1.91 | -27.36±2.93 | 0.34±0.01 |
|  |  |  | 7 | 47.81±2.31 | 4.78±0.23 | 164.22±73.36 | -29.33±4.86 | 0.34±0.01 |
|  |  |  | 8 | 41.44±0.76 | 0.41±0.08 | 211.06±5.55 | -32.17±0.48 | 0.36±0.02 |
|  |  |  | 9 | 39.72±0.21 | 3.97±0.02 | 278.53±22.60 | -32.62±1.04 | 0.41±0.01 |

Table. S3 Amino acid compositions (g/100 g of protein) of RP and RPHs.

| **Amino acid** | **RP** | **RPH(A)** | **RPH(T)** | **RPH(N)** | **RPH(F)** |
| --- | --- | --- | --- | --- | --- |
| Asp | 7.98±0.48 | 9.56±0.74 | 9.81±0.76 | 9.18±0.72 | 10.15±0.67 |
| Glu | 10.13±0.7 | 14.00±0.85 | 15.84±0.85 | 12.89±0.71 | 17.20±0.89 |
| Ser | 12.87±0.44 | 21.45±0.42 | 18.33±0.32 | 13.88±0.26 | 25.70±0.25 |
| Gly | 3.95±0.06 | 7.50±0.11 | 3.63±0.02 | 3.26±0.05 | 3.53±0.01 |
| His | 10.67±0.1 | 9.71±0.24 | 8.78±0.12 | 9.46±0.02 | 9.71±0.12 |
| Arg | 3.26±0.22 | 2.57±0.12 | 1.64±0.14 | 2.89±0.12 | 1.66±0.02 |
| Thr | 7.15±0.21 | 7.65±0.15 | 8.46±0.11 | 6.12±0.11 | 6.26±0.12 |
| Ala | 2.25±0.18 | 3.31±0.10 | 2.50±0.12 | 2.00±0.13 | 2.01±0.12 |
| Pro | 8.56±0.26 | 9.96±0.13 | 7.72±0.13 | 6.91±0.11 | 6.46±0.4 |
| Tyr | 6.73±0.27 | 4.52±0.26 | 5.94±0.14 | 7.06±0.24 | 6.72±0.12 |
| Val | 7.24±0.24 | 7.29±0.17 | 6.48±0.14 | 5.54±0.13 | 4.67±0.24 |
| Met | 2.57±0.15 | 2.20±0.18 | 2.53±0.02 | 2.20±0.06 | 0.90±0.16 |
| IIe | 1.60±0.03 | 1.68±0.02 | 1.45±0.05 | 1.07±0.05 | 1.12±0.23 |
| Leu | 11.72±0.18 | 9.53±0.22 | 9.27±0.04 | 8.36±0.04 | 7.26±0.14 |
| Cys | **Not determined** | | | | |
| Phe | 5.11±0.15 | 4.49±0.21 | 5.30±0.08 | 4.41±0.07 | 4.10±0.05 |
| Lys | 3.99±0.17 | 3.09±0.13 | 3.34±0.19 | 2.69±0.09 | 3.02±0.06 |
| TAAs | 105.78 | 118.51 | 111.02 | 100.83 | 109.47 |
| AAAs | 11.84 | 9.01 | 11.24 | 9.47 | 8.82 |
| HAAs | 40.83 | 55.12 | 52.2 | 43.21 | 59.41 |
| NCAAs | 18.11 | 23.56 | 25.65 | 22.07 | 27.35 |
| EAAs | 56.78 | 50.16 | 51.55 | 44.91 | 41.76 |

The results are expressed as the mean ± standard deviation (n = 3).

Total amino acids (TAAs)

Aromatic amino acids (AAAs): Phe, Tyr.

Hydrophilic amino acids (HAAs): Ser, Thr, Tyr, Glu, Gly.

Negatively charged amino acids (NCAAs): Asp, Glu.

Essential amino acids (EAAs): Thr, Val, Met, Ile, Leu, Tyr, Phe, Lys, His.
